# Local Treatment of Carbuncle

**Published:** 1885-08-20

**Authors:** J. S. Geigley

**Affiliations:** Illinois


### Southern Medical Record.

EDITORS:

THOMAS S. POWELL, M. D. R. C. WORD, M. D.

*R. C. WORD, M.D., Managing Editor.*

☞All Communications and Letters on Business connected with the RECORD must be addressed to the Managing Editor.

VOL. XV. ATLANTA, GA., AUGUST 20, 1885. NO. 8.

### ORIGINAL AND SELECTED ARTICLES.

### LOCAL TREATMENT OF CARBUNCLE.

BY J. S. GEIGLEY, M. D., OF ILLINOIS.

A careful observer of the medical journal literature of the present day cannot help being struck with the variety of opinions expressed therein in regard to the treatment of carbuncle. While one individual will advocate an intricate plan of local treatment, with an extensive paraphernalia of unguents, poultices, and lotions, another will eschew everything but the more heroic surgical measures of incisions, cauterizations, etc., while a third will express nihilistic views in regard to *all* local treatment. Now, there is but one rational conclusion at which he could arrive, from the consideration of these widely different opinions, namely: that their authors were thinking of different things; or, in other words, some of them had treated something for carbuncle that was *not* carbuncle. If we look up the usual text-book definition of carbuncle, we will find it is something like this: a circumscribed indurated inflammation of the skin and subcutaneous cellular tissue, rapidly running into suppuration and slough.

The signs of a carbuncle may be briefly described thus: A pointed pimple, surmounting a broad, flat, indurated swelling, more or less circumscribed, of a brawny red hue, not so exquisitely tender to the touch as a boil, but giving more or less burning and stinging pain. There is, at no time, evidence of fluctuation in the tumor, but it may have somewhat of a doughy feel. In a few days the skin covering the tumor takes on a brownish hue, becomes undermined, a number of small blisters appear, and it gives way at various points, forming openings, from which issue a thin, purulent discharge. The sloughing process now goes on with more or less rapidity, the openings gradually coalesce, and, at the end of four or six weeks, a large granulating ulcer occupies the former site of the tumor.

The above description applies to the evolution of the ordinary carbuncle, as we meet with it in this country, at the present time; but if we accept the statements of writers of the last century, they certainly had to do with a much more formidable affection under that name than we of the present ever encounter; for instance, Benjamin Bell, writing one hundred years ago, says of this affection: "This rapid progress of inflammation occurs most frequently in cases of carbuncle, what by the French is termed *charbon*, in which inflammation proceeds so quickly to mortification that, in some cases, no evident tumor takes place, and the parts become black and completely mortified, often in the course of twenty-four hours from the first attack." It is certainly very rare, now-a-days, that a carbuncle manifests such activity.

We sometimes see carbuncles described, in connection with feruncles or boils, as if they were intimately related, whereas there is nothing in common between them. While a boil is simply a circumscribed cavity containing pus, causing little or no constitutional disturbance, recurring frequently in robust individuals, and readily curable by evacuating the contents, a carbuncle, on the other hand, has no cavity outside of being honey-combed by the sloughing process, causes great constitutional disturbance, occurs generally in persons that are in a debilitated or vitiated state, and only terminates in a slough of all the tissues implicated in the affection. Now, while one might be deceived by a group of boils into making a diagnosis of carbucle, we could hardly fail to be undeceived before the termination of the case. However, I have known of several such "carbuncles," in which remarkable results were obtained by very simple treatment.

As I had no intention of writing a lengthy article on this subject, but simply to preface with a few remarks the report of a case or two, treated somewhat differently from the way I had been in the habit of treating carbuncle, and in which the results obtained seemed to me to justify their publication, I will proceed with the report of my cases:

### CASE I.

F. C——, aged sixty-two, had been confined to his bed for two months with abscess of the liver. Sickness had greatly emaciated him. About the time he began to convalesce, noticed a hard spot on the right buttock, which gave him a great deal of burning pain. I was requested to examine it about a week after it first made its appearance. It had been poulticed for several days previous to my examination, with the expectation of bringing it to a "head." I found a flat, circular, indurated tumor, slightly raised above the surface, of a dark red color, and about four inches in diameter. Careful palpation gave no fluctuation. There were several pimples scattered over the surface of the tumor, which were discharging a thin, watery matter. I made a deep incision through the center of the tumor, but found little or no pus. Ordered a hot linseed-meal poultice to be applied, and renewed frequently. As there was considerable constitutional disturbance, in the way of occasional rigors, and elevations of temperature, I ordered quinine in 5-grain doses every four hours, large doses of a beef wine and iron mixture, and a hypodermic of morphia, morning and evening, to relieve the pain. At the end of a week of this treatment, the tumor presented the following appearance: The skin covering the tumor was a dirty, brownish-red, having several openings, through which a probe could be passed in every direction; in fact, all the tissues composing the tumor were completely honey-combed; small ash-colored sloughs were coming away through the openings, which were rapidly running together through the sloughing process. Thinking to save both time and the patient's strength, I proposed to him to cut away all the sloughing tissue, and convert it into a simple open wound. He consented; and, accordingly, the next day, the patient being anæsthetized, I proceeded to operate by cutting away all the sloughing tissue on either side of the former incision with a strong pair of curved scissors; the parts of the cavity that could not be reached with the scissors was thoroughly scraped with the sharp spoon. After the hemorrhage was checked, the entire raw surface was brushed over with a 95-per cent. solution of carbolic acid, then covered with a thick layer of iodoform; a wad of absorbent cotton was placed in the cavity, a fold of several thicknesses of iodoform gauze was laid over the cotton, and the whole secured by adhesive strips. Patient rested well that night, and, in fact, all the next day. Thirty-six hours after operation removed dressings, which were saturated with pus. After cleansing the wound by irrigating with carbolized water, found that the strong carbolic acid that had been applied had caused quite a slough, and when this had come off the surface looked healthy ; dressed with powdered iodoform, cotton and gauze, as before. These dressings were repeated daily. At the end of a week examination revealed no further sloughing, but, on the contrary, a healthy, granulating surface. At the end of another week the condition of the patient was practically unchanged ; although there was no sloughing, neither was there any cicatrization ; the low condition of the patient's strength, no doubt, prevented the latter. He finally became very impatient to have the sore healed, and suggested that I should try the same treatment that I had used in the case of a friend of his, who had a chronic ulcer of the leg—he referred to the use of sponge grafts. Thinking the suggestion a good one, I concluded to act on it at once.

The sponge was prepared by mascerating in very dilute sulphuric acid, then washed thoroughly, and cut into slices about a quarter of an inch in thickness; this is best done by compressing the sponge in the fingers, and slicing it with a razor or sharp knife. The sponge thus prepared is kept for use in a 5-per cent. solution of carbolic acid.

After carefully cleansing the wound, which was now about three inches in diameter, a piece of the sponge was squeezed out of the solution, the edges were trimmed to approximate the dimensions of the ulcer, and, after immersing in the etherial solution of iodoform, 20 grains to the ounce, it was carefully placed on the raw surface, a piece of folded iodoform gauze was laid over the sponge, then a thick sheet of absorbent cotton, followed by gutta percha tissue, and the whole secured by adhesive strips and a bandage. First examination, two days afterwards, the gauze had absorbed considerable pus, and there was a good deal about the wound. I cleansed it by irrigating with carbolized water, and with wads of absorbent cotton. I found the sponge adherent at one edge only, in about a quarter of its circumference ; the discharges were perfectly sweet, and I pressed the sponge in place, and dressed as before. The next examination, two days afterward, showed fully a third of the sponge adherent ; the loose part I snipped away with scissors, as it did not look to be in a satisfactory condition. The discharge had now materially diminished, the diameter of the ulcer was lessened, and there was quite a change in the appearance of things generally. On the 15th, or eighteenth day from the application of the graft, small points of granulation could be seen springing through the meshes of the sponge ; by the twenty-fifth day the surface of the ulcer was nearly level with the surrounding skin, and was cicatrizing rapidly. On the fortieth day the sponge had entirely disappeared.

The general condition of the patient during this time was carefully looked after. He had a generous diet, with tonics, stimulants, and opiates, as they were indicated.

Now, while the time occupied in the cicatrizing process in this case would not be considered short in a healthy individual, at first thought one might be inclined to question the utility of the sponge graft; but, when we take into consideration the patient's age, his state of health at the beginning of the morbid process, then the inactivity of the granulating process for several weeks after the operation, it seems to me, we cannot help concluding that the sponge materially quickened the cicatrizing process.

CASE II.

P. R——, farmer, aged fifty-three, had been having "third-day ague" for a year and a half; had also been suffering for some time past with a purulent bronchial catarrh. About two weeks before I was called, he noticed what he supposed was a "little boil" low down on the back of his neck. This discharged a little at first, but the tissues for two inches around the base soon became inflamed and hard, giving him an intense burning and lancinating pain, so that he could not sleep. A bread-and-milk poultice was applied for several days, but this gave him very little relief. In a week or ten days four or five openings were observed, all discharging slightly. His general condition becoming rapidly worse, at the end of two weeks I was called. Examination revealed a sloughing mass of indurations, extending from the seventh cervical vertebra well over on the right shoulder. The tumor was about four inches in diameter, and completely honey-combed. Pressure would cause an unhealthy looking pus to ooze from the openings. His general condition was very bad, and the thermometer in the axilla gave a temperature of 102°. I immediately ordered large doses of quinine, to control the malarial element in the case, and gave hypodermics of morphia, as they were needed to relieve pain; also, ordered a liberal allowance of egg-nog, and beef wine and iron. A hot linseed meal poultice was kept on the tumor; and that night the patient rested well. Next day, the temperature being nearly normal, and the general condition much improved, I proposed to excise the tumor completely; patient consented, and, after having him completely anæsthetized, I proceeded by making a deep cut through the center of the sloughing mass, and attacking the tissues on either side with volsella forceps, scissors, and sharp spoon. After the sloughing tissue was gotten rid of, the strong carbolic acid was applied as in the other case; the dressings were the same also. In four days from the operation the wound was clean, and I determined to try the sponge grafts again, as the wound was so large (about four inches in diameter). I had to use two pieces of sponge to fill it completely. Now, as I do not wish to go into details in this case, it will suffice to say that one of the grafts and part of the other was entirely adherent inside of ten days; the one that entirely adhered was completely absorbed, while the other came off about the twentieth day through the meddling of an officious nurse. Cicatrization was complete in the case inside of two months from the operation.

The advantages of the plan pursued in the above cases are, briefly, these: 1. The removal of the involved tissues quickly by surgical means, instead of waiting for the slow and exhausting process of removal by slough; 2. The promotion of cicatrization in the wound by the use of sponge grafts, thereby insuring rapid cicatrization with little or no liability to contractions in the cicatrix.

